# Recombinant frizzled1 protein attenuated cardiac hypertrophy after myocardial infarction via the canonical Wnt signaling pathway

**DOI:** 10.18632/oncotarget.23149

**Published:** 2017-12-12

**Authors:** Jingjing Fan, Lin Qiu, Hongyang Shu, Ben Ma, Marco Hagenmueller, Johannes H. Riffel, Soeren Meryer, Min Zhang, Stefan E. Hardt, Lin Wang, Dao Wen Wang, Hongyu Qiu, Ning Zhou

**Affiliations:** ^1^ Division of Cardiology, Department of Internal Medicine, Tongji Hospital, Tongji Medical College, Huazhong University of Science and Technology, Wuhan, P.R. China; ^2^ Department of Cardiology, University of Heidelberg, Heidelberg, Germany; ^3^ Department of Pharmacy, Tongji Hospital, Tongji Medical College, Huazhong University of Science and Technology, Wuhan, P.R. China; ^4^ Division of Physiology, Department of Basic Sciences, School of Medicine, Loma Linda University, Loma Linda, CA, USA

**Keywords:** frizzled1, cardiac hypertrophy, myocardial infarction, wnt signaling pathway, recombinant protein

## Abstract

Postinfarct cardiac hypertrophy is an independent risk factor for heart failure and sudden death. Regression of cardiac hypertrophy has emerged as a promising strategy in the treatment of myocardial infarction (MI). Here we hypothesized that frizzled1 (FZD1), a receptor of the canonical Wnt signaling pathway, is a novel mediator of ischemia-associated cardiac hypertrophy. MI was induced in mice by left anterior descending (LAD) coronary occlusion. One week after MI, the expression of FZD1 was found to be notably increased in the left ventricles (LVs) of the MI-mice compared to shams. Mouse recombinant FZD1 protein (RFP) was subcutaneously injected in the mice to provoke autoimmunization response. Anti-FZD1 antibody titer was significantly increased in the plasma of RFP-treated mice. RFP significantly mitigated the MI-induced cardiac hypertrophy and improved cardiac function in the MI mouse hearts. Moreover, increased heart and LV weights, myocardial size and the expression of β-myosin heavy chain in the MI-mice were also found to be attenuated by RFP. FZD1 was found to be significantly up-regulated in hypoxia-treated neonatal rat cardiomyocytes (NRCMs). Silencing FZD1 by siRNA transfection notably repressed the hypoxia-induced myocardial hypertrophy in NRCMs. Mechanistically, activation of canonical Wnt signaling induced by MI, e.g., β-catenin and glycogen synthase kinase-3β, was restrained in the LVs of the MI-mice treated by RFP, these inhibition on canonical Wnt signaling was further confirmed in hypoxic NRCMs transfected with FZD1 siRNA. In conclusion, immunization of RFP attenuated cardiac hypertrophy and improved cardiac function in the MI mice via blocking the canonical Wnt signaling pathway.

## INTRODUCTION

Myocardial infarction (MI) is a major cause of cardiovascular morbidity and mortality which has drawn extensive attention for decades [1]. As an independent risk factor of sudden cardiac death and heart failure, post-MI cardiac hypertrophy has emerged as a promising target in the treatment of MI patients [2]. Besides traditional medications including the β-adrenergic receptor blockers and angiotensin converting enzyme inhibitors, new strategies seeking to mitigate the cardiac hypertrophy have been arising , such as local injection or transplantation of progenitor cells, growth factors or genes [3, 4]. Artificially active immunization targeting the pro-hypertrophic factors has been identified as a novel strategy for the treatment of cardiac hypertrophy after MI [5]. However, the effective targets as well as the safe and efficient methods of activating immunization remain undetermined so far.

The Wnt family of secreted glycoproteins is involved in a wide array of biological processes, including cellular survival, apoptosis, differentiation, proliferation, angiogenesis and hypertrophy [6, 7]. The canonical Wnt signaling primarily promotes β-catenin-mediated activation of transcription, while the non-canonical Wnt signaling involves a calcium-dependent protein kinase C-mediated Wnt/Ca^2+^ pathway and a dishevelled-dependent c-Jun N-terminal kinase-mediated planar cell polarity pathway [8, 9]. Although both canonical and non-canonical Wnts have been implicated in various cardiac responses to physiological and pathological stimuli [10, 11], canonical Wnt signaling plays dominant roles in the myocardial regeneration, specification, morphogenesis and differentiation [9, 12]. It has been showed that blocking of Wnt3a-associated signaling could prevent the development of heart failure after MI [13]. Upon pathological stress, Wnt3a binds to its receptor, frizzled1 (FZD1), a seven-transmembrane receptor abundantly expressed in the heart tissue, increased the phosphorylation of GSK-3β and consequently blocked the β-catenin degradation [14]. Therefore, FZD1 has merged as a potential therapeutic target to improve the prognosis of the MI patients [13, 15]. However, the safe and effective approach to inhibit FZD1 *in vivo* is still far from resolved. Increasing evidences showed that immunotherapy is a potential therapeutic approach of cardiac hypertrophy [16]. Thus, we hypothesized that targeting the FZD1 may open new pharmacological venues for treating cardiac hypertrophy and heart failure after MI to supplement current drugs that target the sympathetic or renin-angiotensin systems.

In the present study, by using an MI mouse model and hypoxia-treated NRCMs, we unravelled a notable attenuation of cardiac hypertrophy after MI led by the immunization of RFP through blocking canonical Wnt signaling pathway. Our findings may provide an optional therapeutic strategy to inhibit pathological cardiac remodelling and improve the cardiac function after MI.

## RESULTS

### FZD1 was up-regulated by both MI and hypoxic stimuli

**Figure 1:**
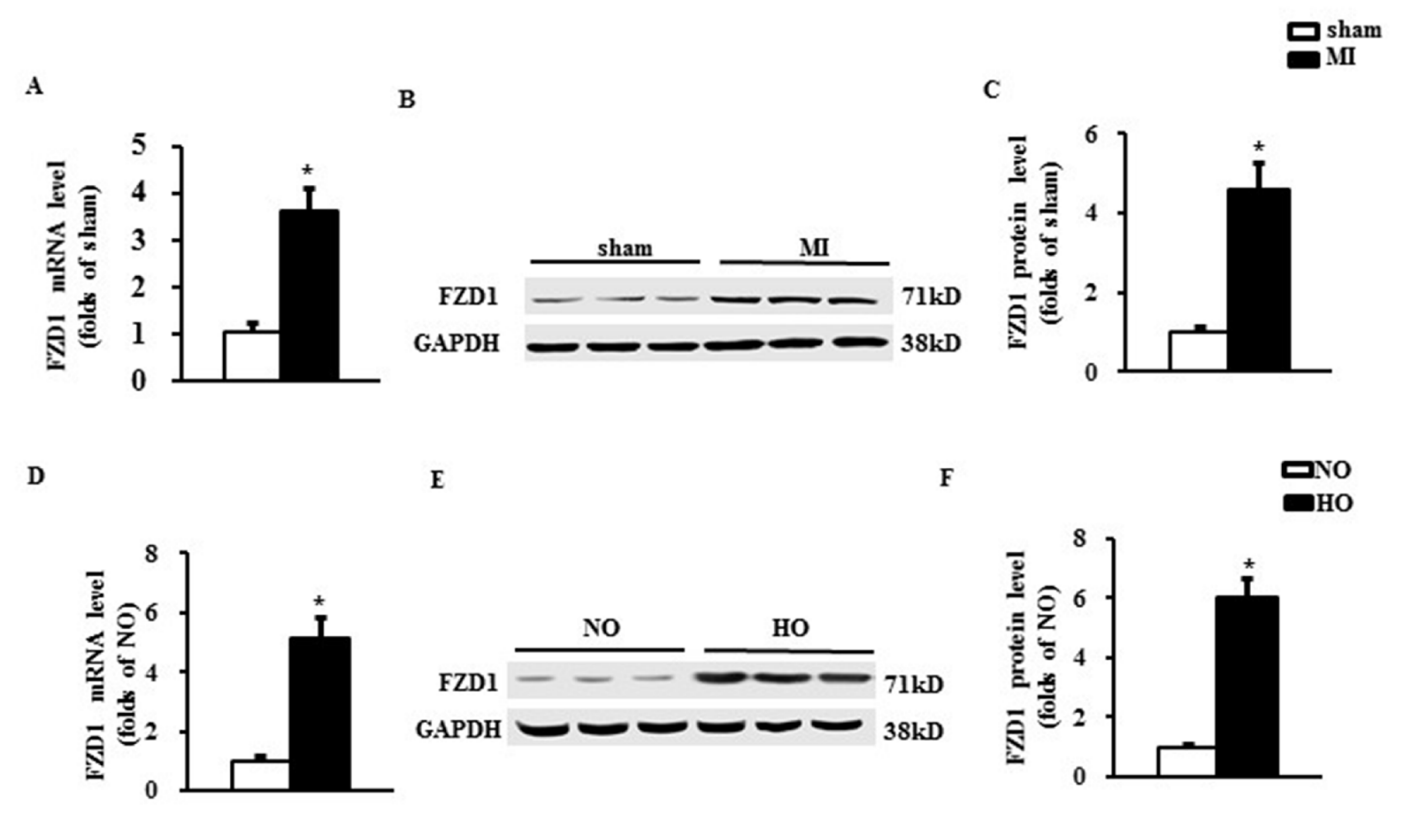
FZD1 was up-regulated by both MI and hypoxic stimuli (**A**) mRNA level of FZD1 in the non- infarct mouse left ventricles (LVs) one week after MI. HPRT was served as internal control. (**B**) Representative Western blots and (**C**) quantitative results of FZD1 in the LV one week after MI. *N* = 5 per experimental group. ^*^P < 0.01 compared with sham. (**D**) mRNA levels of FZD1 in neonatal cardiomyocytes (NRCMs) stimulated by hypoxia. HPRT was served as internal control. (**E**) Representative Western blots and (**F**) quantitative results of FZD1 in NRCMs stimulated by hypoxia. *N* = 5 independent experiments. GAPDH was served as internal control. NO, normoxia. HO, hypoxia. All data are expressed as mean ± S.E.M. ^*^P < 0.01 compared with NO.

To identify the relationship between FZD1 and ischemic cardiac hypertrophy, we measured the expression of FZD1 in cardiomyocytes *in vivo* and *in vitro*. First, an MI model was induced in mouse hearts by left anterior descending (LAD) coronary occlusion and the expression of FZD1 was detected in the infarct border zone of the mouse LVs at one-week post-MI when the cardiac hypotrophy has been developed. As shown in Figure 1A to 1C, FZD1 was found to be significantly increased in both mRNA and protein levels by 3.6-folds and 4.6-folds, respectively, in the infarct border zone of the mouse LVs compared to sham mice. We also measured the expression of FZD1 in other organs including brain, lung, liver and muscle after MI and no significant changes on the FZD1 expression were found before and after MI in the above mouse organs (Supplementary Figure 1). Second, to eliminate the possible compensatory or neuroendocrine factors which may influence the expression of FZD1 in the MI-induced cardiac hypertrophy, isolated neonatal rat cardiomyocytes (NRCMs) were stimulated by hypoxia, to mimic MI in an *in vitro* study. The mRNA and protein levels of FZD1 were increased by 5.2-folds and 6.0-folds, respectively, in the hypoxia-treated NRCMs compared to the normoxia-treated NRCMs (Figure 1D to 1F).

### Deficiency of FZD1 alleviated hypoxia-induced myocardial hypertrophy *in vitro*

**Figure 2:**
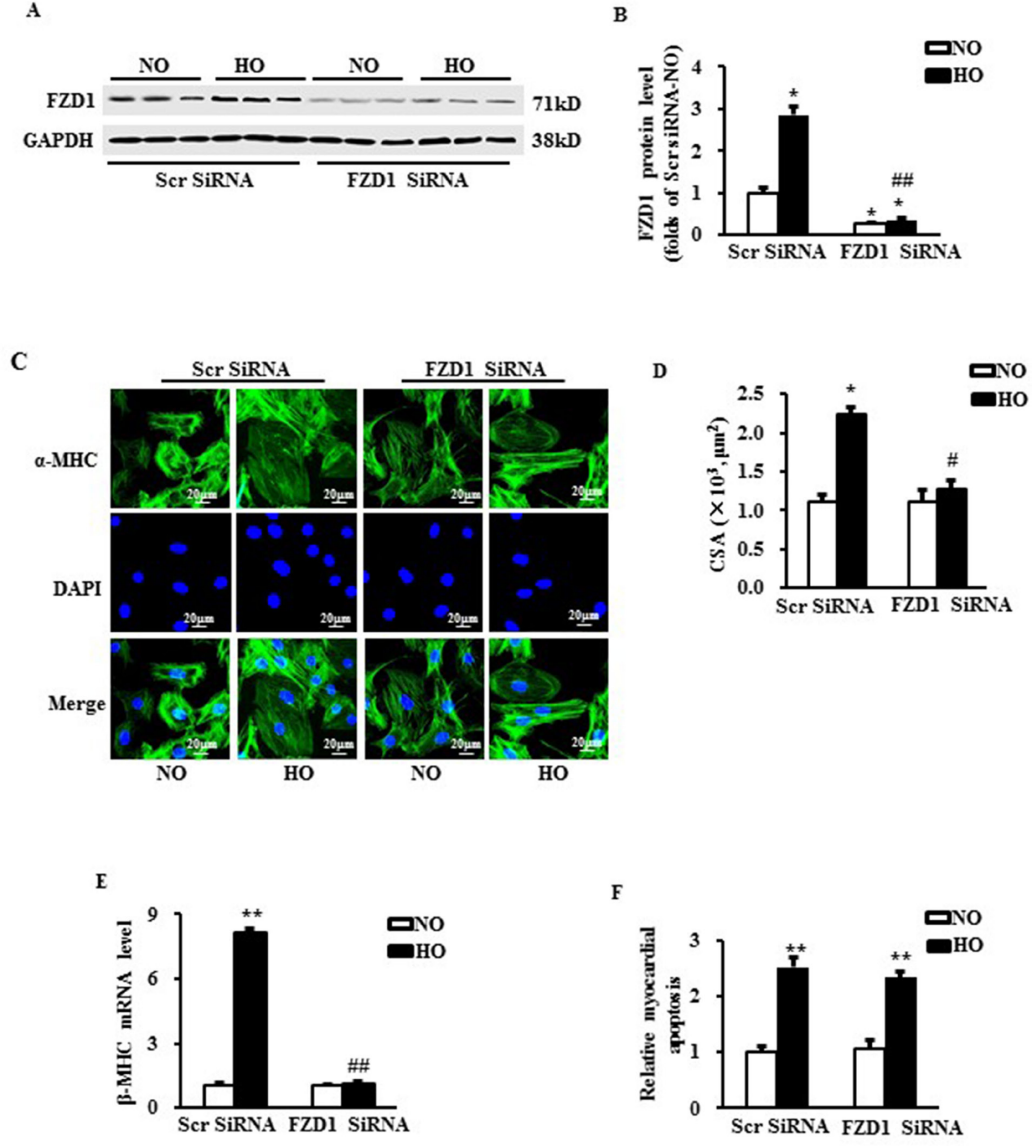
Deficiency of FZD1 alleviated hypoxia-induced myocardial hypertrophy *in vitro* (**A**) Representative Western blots of FZD1 in neonatal cardiomyocytes (NRCMs) after transduced with indicated SiRNA and treated with hypoxia. *N* = 5 independent experiments. (**B**) Quantitative results of the protein expression of FZD1in NRCMs. (**C**) The representative images of NRCMs stained by α- myosin heavy chain (α-MHC). (**D**) The quantification data of NRCM surface area (CSA). (**E**) mRNA levels of hypertrophic markers, β-myosin heavy chain (β-MHC) in NRCMs measured by real-time PCR. HPRT was served as internal control. (**F**) The myocardial apoptosis expressed as the ratio of TUNEL-positive nuclei over DAPI-stained nuclei. *N* = 5 independent experiments. GAPDH was served as internal control in Western blot. NO, normoxia. HO, hypoxia. All data are expressed as mean ± S.E.M. ^*^P < 0.05 compared with NO + Scr SiRNA, ^#^*P* < 0.05 compared with HO + Scr SiRNA.

Given upregulation of FZD1 in hypertrophic hearts under myocardial ischemia, we tested whether an decrease of FZD1 expression prevents cardiac hypertrophy *in vitro*. NRCMs were exposed in hypoxic condition for 24h. FZD1 siRNA was transfected in NRCMs to knockdown the expression of FZD1 in the cardiomyocytes (Figure 2A). We found that hypoxia notably increased the expression of FZD1 in control NRCMs, but not in NRCMs with the transfection of FZD1 siRNA (Figure 2A and 2B). The NRCMs were then subjected to immunostaining using α-Myosin heavy chain (α-MHC) primary antibody as shown in Figure 2C. Hypoxia induced a 2.2-folds increase in the cardiomyocyte surface area (CSA) of NRCMs (Figure 2D), and an 8.1-folds increase in mRNA level of β-Myosin heavy chain (β-MHC), a myocardial hypertrophy-associated marker (Figure 2E). Transfection of FZD1 SiRNA significantly repressed the increase of CSA and the upregulation of β-MHC (Figure 2D and 2E). TUNEL assay was performed to detect the myocardial apoptosis. Hypoxia induced a 2.5-folds increase in the cell apoptosis in NRCMs compared to normoxic NRCMs, which was not alterated by the treatment of FZD1 SiRNA (Figure 2F). Together, these data indicated that reduction of FZD1 attenuated hypoxia-induced myocardial growth *in vitro* rather than cell death, indicating a role of FZD1 in the hypoxia-induced myocardial hypertrophy.

### Treatment with RFP provoked auto-immunization in mice

**Figure 3:**
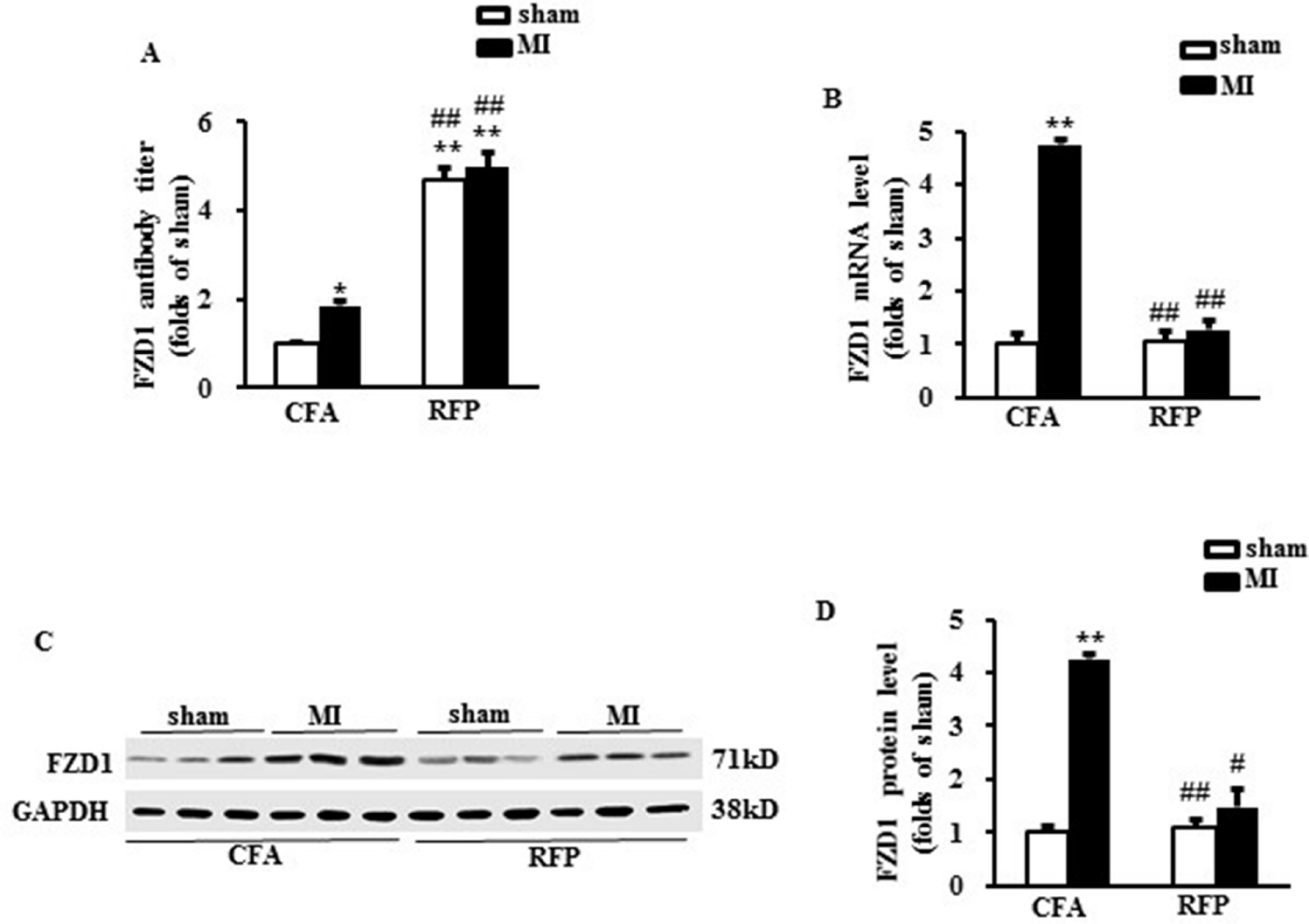
Treatment with RFP provoked auto-immunization in mice (**A**) The FZD1 auto-antibody titer in mouse plasma. (**B**) Real-time PCR analysis of FZD1 in mouse left ventricles (LVs) after MI. HPRT was served as internal control. (**C**) Representative Western blots and (**D**) the quantitative results of FZD1 in mouse LVs one week after MI or sham surgery. GAPDH was served as internal control in all Western blot. All data are expressed as mean ± S.E.M. ^*^P < 0.05, ^**^P < 0.01 compared with sham + CFA, ^#^*P* < 0.05, ^##^*P* < 0.01 compared with MI + CFA.

To provoke auto-immunization response against FZD1, mice were subcutaneously injected with mouse RFP for twice, one at the 1st day and a repeated one at 7th day of the study. The mice were subjected to LAD or sham operation at 21th day after the first injection. Then the mice were sacrificed one week after surgery. The mouse plasma was collected to measure the concentration of FZD1 autoantibody. Treatment with RFP dramatically increased the plasma concentration of specific FZD1 autoantibody by 4.7-folds and 4.9-folds in FZD1-treated sham and MI mice, respectively, implying a successful autoimmune response in mice (Figure 3A). The expression of FZD1 in the infarct border zone of the mouse LVs was significantly decreased in RFP-treated MI mice on both mRNA and protein levels (Figure 3B to 3D) compared to vehicle treated MI mice. In sham-operated mice, the expression of FZD1 was unchanged after the treatment of RFP (Figure 3B to 3D). In addition, treatment with RFP had no significant effect on the expression of FZD1 in the brain, lung, liver and muscle in the MI mice (Supplementary Figure 2). Collectively, subcutaneously injection with RFP provoked remarkable auto-immunization which specifically repressed the expression of endogenous myocardial FZD1 in MI mice.

### RFP attenuated cardiac hypertrophy and improved cardiac dysfunction after MI

Since RFP was able to repress the expression of endogenous myocardial FZD1 in the MI mice, we next interrogated whether such a reduced myocardial FZD1 expression could attenuate the MI-induced cardiac hypertrophy and dysfunction. First, cardiac morphology and function of the mice were measured by echocardiography one week after LAD ligation. The quantitated data showed that there was no significant difference between the sham vehicle-treated mice and RFP-treated mice in terms of cardiac morphology and contractile function (Table 1), indicating that RFP did not affect cardiac development and growth under physiological condition. One week after MI, vehicle-treated mice developed a significant left ventricular hypertrophy compared to sham mice, represented by a significant increase in LV wall thickness (left ventricular anterior wall end-diastolic thickness, LVAWD) , a notable decrease in left ventricular ejection fraction (EF) and preserved heart rate (HR), left ventricular internal end-diastolic dimensions (LVIDd) (Table 1). In addition, the MI-induced hemodynamic alteration was measured by invasive cardiac catheter. Significant decrease of LV end-systolic pressure (LVESP) and increase of LV end-diastolic pressure (LVEDP) were observed in the MI mice compared to the sham mice (Table 1). Although the LVEF and LVEDP of MI mice treated with RFP were worsened compared to the sham mice, these alterations were significnatly mitigated versus MI mice treated by vehicle. Furthermore, the MI mice also showed a significant decrease of maximal contraction and relaxation velocity (max dp/dt and min dp/dt) versus sham, while these hemodynamic alterations were also improved in RFP-treated MI mice (Table 1) .

**Table 1:** Echocardiographic and hemodynamic analysis of mice one week after sham or LAD ligation

	Sham		MI	
	Vehicle (*n* = 5)	RFP (*n* = 5)	Vehicle (*n* = 5)	RFP (*n* = 5)
LVAWd (mm)	0.72 ± 0.03	0.74 ± 0.04	1.19 ± 0.05^**^	0.79 ± 0.05^#^
LVIDd (mm)	3.04 ± 0.1	3.11 ± 0.2	3.94 ± 0.3^**^	3.18 ± 0.3^*#^
EF (%)	75.5 ± 3.4	76.1 ± 5.5	58.2 ± 2.5^**^	66.5 ± 3.7^*##^
HR (bpm)	551.2 ± 44.4	567.5 ± 53.1	571.2 ± 47.2	561.2 ± 41.8
LVESP (mmHg)	98.2 ± 6.4	97.1 ± 5.5	82.6 ± 3.2^**^	92.6 ± 6.9^##^
LVEDP (mmHg)	1.7 ± 0.1	1.8 ± 0.1	7.7 ± 0.4^**^	3.5 ± 0.3^*#^
Max dp/dt (mmHg/S)	10116 ± 564.5	9946 ± 411.5	6018 ± 376.1^*^	8287 ± 506.3^*#^
Min dp/dt (mmHg/S)	−8876 ± 334.6	−8344 ± 504.1	−5044 ± 207.6^*^	−6944 ± 343.8^*#^

Note: LVAWd, left ventricular anterior wall end-diastolic thickness; EF, ejection fraction; LVIDd, left ventricular internal end-diastolic dimensions; HR, heart rate; LVESP, LV end-systolic pressur; LVEDP, LV end-diastolic pressure; max dp/dt, maximal contraction velocity; min dp/dt, maximal relaxation velocity; ^*^p < 0.05, ^**^p < 0.01 vs. respective sham mice. ^#^*p* < 0.05, ^##^*p* < 0.01 vs. MI-vehicle mice.

**Figure 4:**
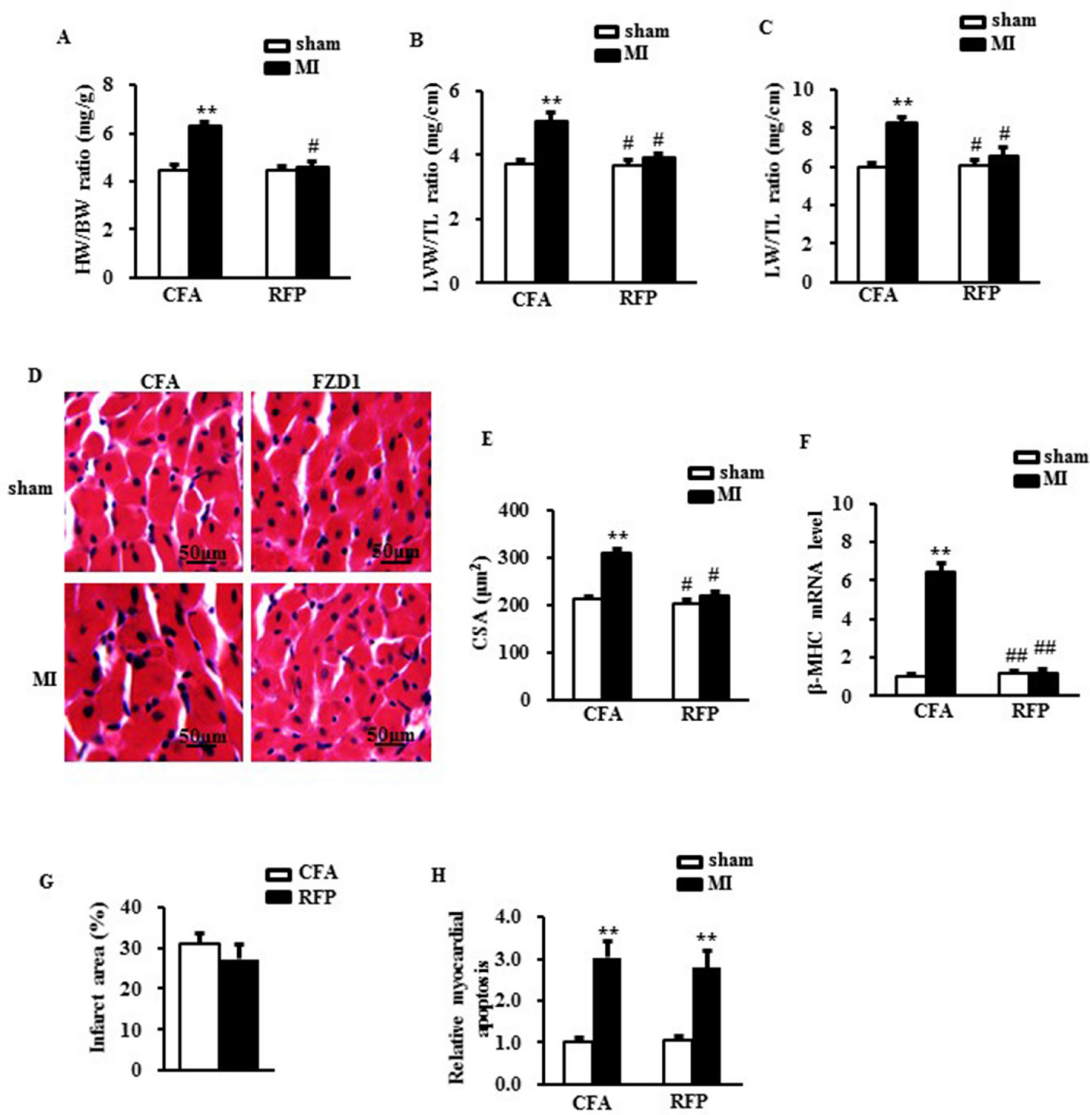
RFP attenuated cardiac hypertrophy and rescued cardiac dysfunction after MI (**A**) Heart weight/ body weight ratio (HW/BW), (**B**) Left ventricular weight/ tibia length ratio (LVW/TL) and (**C**) Lung weight/ tibia length ratio (LW/TL) of mice treated by indicated surgery and treatments. (**D**) Representative images of histological sections of the mouse LVs were stained with hematoxylin-eosin (H&E) one week after MI or sham surgery. Scale bar: 50 μm. (**E**) Quantitative results of the cross sectional area (CSA) of mouse cardiomyocytes quantified by using an image analysis system. (**F**) Real-time PCR analysis of β-MHC in mouse LVs after MI. HPRT was served as internal control. (**G**) The relative infarcted area of mouse left ventricles. (**H**) the relative ratio of myocardial apoptosis in the infarct border zone of the mouse left ventricles. *N* = 5 per experimental group. CFA, Complete Freund’s adjuvant. *N* = 5 independent experiments. All data are expressed as mean ± S.E.M. ^**^P < 0.01 compared with sham + CFA, ^#^*P* < 0.05, ^##^*P* <0.01 compared with MI + CFA.

Cardiac hypertrophy was further determined by the direct *ex vivo* measurements and the histological analysis in the heart tissues one week after MI. Compared to the sham mice, the MI mice exhibited a significant increase in heart weight/body weight ratio (HW/BW) (Figure 4A), LV weight/tibal length ratio (LVW/TL) (Figure 4B), lung weight/ tibia length (LW/TL) (Figure 4C) and cross-sectional area (CSA) of cardiomyocytes (Figure 4D and 4E), whereas these alterations were significantly mitigated by tretament of RFP (Figure 4A to 4D). Additionally, cardiac hypertrophic markers, β-MHC was significantly elevated in the MI mice versus sham mice which were reversed by RFP (Figure 4F). To reveal whether the attenuation of cardic hypertophy was a resultant change of the myocardial infaction, we measured the infarct size of the mouse hearts. As shown in Figure 4G, there is no significant difference in the infarct size between RFP-treated mice and vehicle-treate mice. We also detected the MI-induced myocardial apoptosis using TUNEL assay. There was a remarkable increase in the myocardial apoptosis in the infarct border zone of the LVs in the MI mice compared to sham mice. This MI-induced cell apoptosis was slightly attenuated by the RFP (*P* > 0.05, Figure 4H). Collectively, these *in vivo* data, consistent with the observations *in vivo*, further supported the antihypertrophic effect of RFP in the early stage of MI.

### RFP inhibited the canonical Wnt signaling pathway

**Figure 5:**
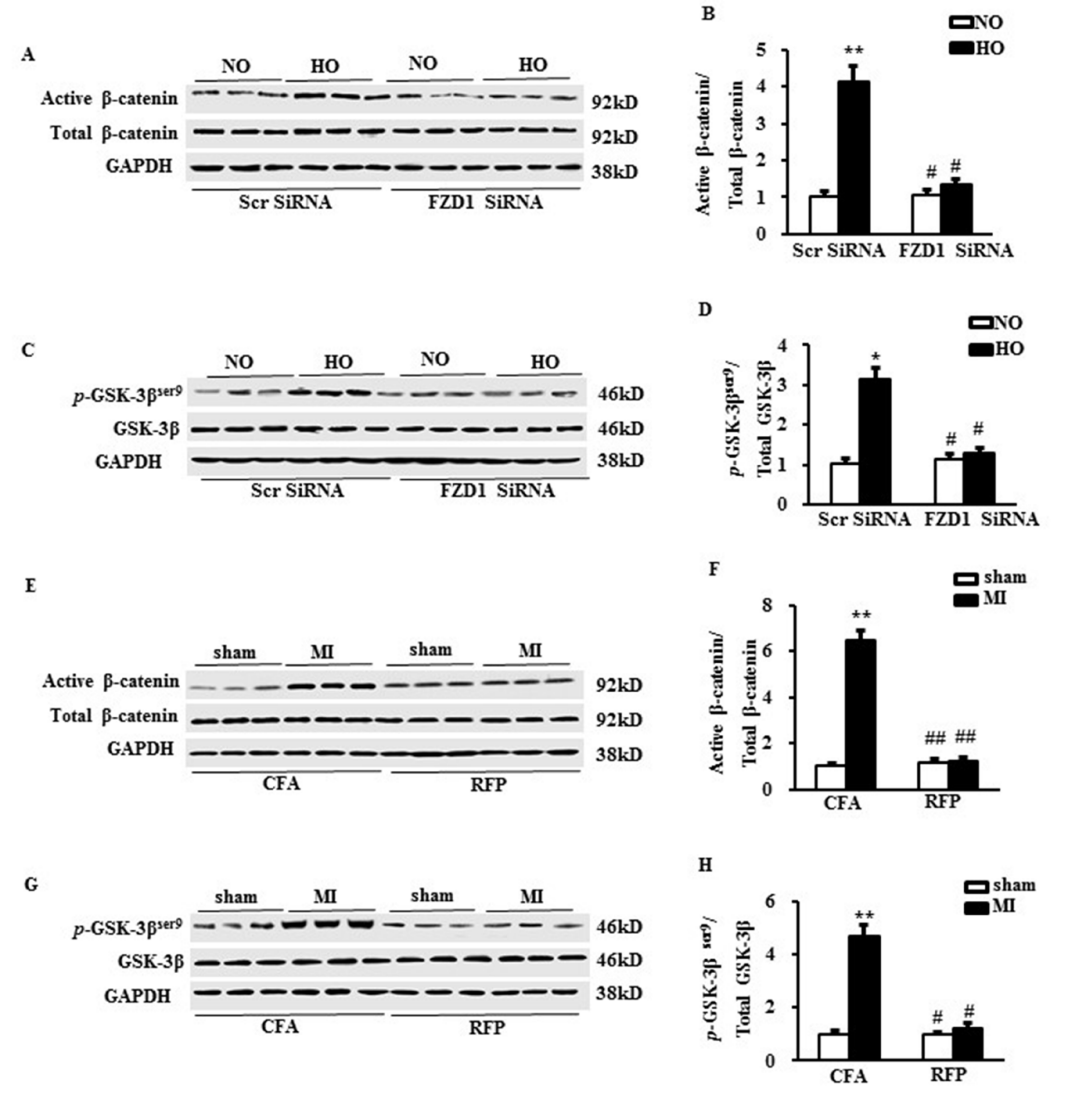
RFP inhibited the canonical Wnt signalling pathway (**A**) Representative Western blots and (**B**) the quantitative result of active/total β-catenin in neonatal cardiomyocytes (NRCMs) after transduced with indicated SiRNA and treated with hypoxia. (**C**) Representative Western blots and (**D**) the quantitative result of phosphorylated/total GSK-3β in neonatal cardiomyocytes (NRCMs) after transduced with indicated SiRNA and treated with hypoxia. *N* = 5 independent experiments in all groups. ^*^P < 0.05, ^**^P < 0.01 vs NO + Scr SiRNA, ^#^*P* < 0.05 vs HO + Scr SiRNA. (**E**) Representative Western blots and (**F**) the quantitative result of active/total β-catenin in mouse left ventricles (LVs) one week after MI or sham surgery. (**G**) Representative Western blots and (**H**) the quantitative result of phosphorylated /total GSK-3β in mouse left ventricles (LVs) one week after MI or sham surgery. *N* = 5 per experimental group. All data are expressed as mean ± S.E.M. ^**^P < 0.01 compared with sham + CFA, ^#^*P* <0.05, ^##^*P* < 0.01 compared with MI + CFA.

To unravel the molecular mechanisms of the anti-hypertrophic effect of RFP, we measured the activation of downstream canonical Wnt signaling, a central pathway mediating cardiac hypertrophy [15, 17]. Both the active β-catenin and phosphorylated glycogen synthase kinase-3β (GSK-3β) at ser9 were significantly increased in the hypoxia-treated NRCMs, which were abrogated by knockdown of FZD1 (Figure 5A to 5D). We then asked whether the treatment of RFP could block the activation of canonical Wnt signaling evoked by MI. As shown in Figure 5E to 5H, increased active β-catenin and the phosphorylation of GSK-3β at ser9 were also inhibited by RFP in ischemic LV tissue *in vivo*. These findings demonstrated that immunization of FZD1 by RFP inhibited the activation of canonical Wnt pathway *in vivo*.

## DISCUSSION

Although cardiac hypertrophy in response to ischemia was considered as an adaptive response to the loss of working cardiomyocytes, this view has been challenged by evidence that the MI-induced LVH consistently damaged the compliance of the hearts and aggravated myocardial ischemia [18]. Cardiac hypertrophy is not only a predictor but also a mediator of cardiovascular events predisposing patients to arrhythmias and heart failure [19]. Therefore, prevention of cardiac hypertrophy is considered as a promising strategy in the treatment of MI [20]. To our knowledge, this is the first report that immunization of FZD1 with RFP attenuated cardiac hypertrophy occurred in the early stage of MI. In the present study, we identified that FZD1 is an essential mediator of cardiac hypertrophy induced by hypoxic stimuli. Our data not only demonstrated a positive correlation between the expression of FZD1 and the cardiac hypertrophy in both cultured-cardiomyocytes *in vitro* and MI animal model *in vivo*, but also showed that reduction of FZD1 using SiRNA resulted in a marked attenuation of hypertrophic response to hypoxia *in vitro*. We further showed that recombinant mouse FZD1 protein notably attenuated cardiac hypertrophy and improved cardiac function *in vivo*, which is independent on the effect of myocardial survival. These findings collectively indicated that inhibition of FZD1 by RFP presents a promising therapeutic potential for the MI patients.

In addition, we revealed that the protective role of RFP on the development of cardiac hypertrophy is associated with the inhibition of the canonical Wnt/GSK-3β/β-catenin signalling pathway. Our study provides insights into the mechanisms of the MI-induced cardiac hypertrophy and dysfunction. FZD1, a seven-transmembrane receptor abundantly expressed in the heart tissue has been shown to be critically involved in the post cardiac ischemia remodelling [21]. It is known that FZD1 binds its ligand Wnt3a and activates the canonical Wnt pathway [22]. Once FZD1 was activated, the downstream GSK-3β is phosphorylated and β-catenin degradation is repressed [23]. Active β-catenin accumulates in the cytoplasm and travels into the nucleus where it activates the expression of Wnt target genes. Although the role of canonical Wnt pathway in MI and cardiac hypertrophy remains controversial, the long championed view is that the canonical Wnt pathway is a key mediator of cardiac hypertrophy induced by MI [14, 15]. It has been showed that deactivating canonical Wnt pathway via increased degradation of β-catenin attenuates the pathological cardiac hypertrophy in mice one week after MI [24]. Our data showed that MI or hypoxia induced an activation of canonical Wnt pathway, while knockdown or treated with RFP significant inhibited the activity of this pathway, indicating a regulatory effect of FZD1 on Wnt signalling under the hypoxia stress in cardiomyocytes. Our results also indicated that activation of FZD1/Wnt signaling plays an essential role in the development of cardiac hypotrophy in the post-MI, and that RFP may provide an interesting therapeutic strategy to reduce the endogenous expression of FZD1, thus protects against cardiac hypertrophy.

Antibodies have been used to reduce the expression of cancer-related antigen and considered as a standard component of cancer therapy for decades. Our results indicated that recombined FZD1 is able to induce antibody that acts as an inhibitor to repress the expression of endogenous FZD1 in the ischemic heart. It is essential that immune-cell could recognize the antigens to trigger the immune response. In the present study, we showed that mice were subcutaneously injected with RFP successfully induced specific antibody against FZD1, indicating that FZD1 is immunogenic in body. Immunocytes recognized RFP as an alloantigen and presented to T-cells and B-cells. Once activated by RFP, B-cells are able to produce specific antibodies. Additionally, once RFP was injected repeatedly, B-cells can produce more antibodies due to a memory effect which quickly and dramatically shortened the time required to mount an immune response [25]. In the present study, a robust increase of plasma concentration of anti-FZD1 antibody was expectedly observed after injections of RFP. Furthermore, unlike intracellular proteins, FZD1, as a transmembrane receptor, can be easily bound to circulating antibodies. Abundant anti-FZD1 antibodies evoked by RFP bound to the transmembrane receptor FZD1 in the ischemic heart tissue, resulting in an inhibition of the endogenous expression of FZD1, which subsequently repressing the Wnt signalling, and thereby supress the cardiac hypertrophy. It is notable that the immune response between FZD1 and auto-antibodies induced by RFP presents a highly tissue specificity in heart and thus provides safer effect with less side effects. Moreover, the inhibition of hypertrophy may also provide a longer-lasting effect than the sole injection of antibody because of the “memory effect” of the immune response. Therefore, these characteristics of FZD1 immunotherapy make it an effective strategy to treat cardiac hypertrophy after MI. Although a remarkable reduction in the expression of the myocardial FZD1 was found in the present study, the underlying mechanism is still unclear yet. Autoantibodies functioned through various mechanisms, including directly targeting the antigen protein, modifying the host response, delivering cytotoxic moieties and retargeting cellular immunity towards the cells [26]. Characteristics of autoantibodies that affect their efficacy include antigen specificity, overall structure, and affinity for the target antigen will improve the autoimmune therapy in cardiovascular disease. Our results lead to a research interest in further mechanistic investigation in REP. Additionally, although we selectively focused on the effect of FZD1 on the canonical Wnt signalling pathway since FZD1 is known as the natural receptor of Wnt3a, it might also possible that FZD1 is involved in other mechanisms depressing pathological hypertrophy that are not known so far. Despite these limitations, our study reveals an important potential of REP in inhibiting the MI-induced cardiac hypertrophy.

In summary, our study brought a novel therapy of immunization of FZD1 to alleviate cardiac hypertrophy induced by MI. We provided evidences illustrating that the underlying cardioprotective mechanism of RFP is mediated by the inhibition of canonical Wnt pathway. The present study indicated a potential target for pharmacological treatment of cardiac hypertrophy after MI in patients.

## MATERIALS AND METHODS

### Isolation and treatment of cardiomyocytes

Primary NRCMs were prepared as described previously [27]. Briefly, 1- to 2-day-old Wistar rats were sacrificed by swift decapitation and hearts were immediately removed. The hearts were minced on ice and digested with trypsin (10 µg/ml). The cells were collected by low-speed centrifugation. The cell pellet was resuspended in Dulbecco’s Modified Eagle Medium (DMEM) containing 10% fetal calf serum (FCS, Hyclone Laboratories, USA). Dispersed cells were pre-plated for 90 min to remove fibroblasts and other proliferation cells, and unattached cells counted and seeded onto 6-well culture plates. Isolated cardiac myocytes were cultured and transfected with either FZD1 or non-specific Scrambled (Scr) siRNA as described before [28]. SiRNAs were commercially purchased from MWG (Ebersberg, Germany). Seventy-two hours after siRNA transfection, myocytes were further cultured under either normoxic (37°C, 5% CO_2_) or hypoxic (37°C, 1.5 % O_2_) condition for 24 h. Immunofluorescent staining for NRCMs was performed using anti-α-MHC) primary antibody and 4′,6-Diamidino-2-Phenylindole, Dihydrochloride (DAPI) staining for 30 min as described previously [29]. Myocyte surface area from at least 100 cells per group in each experiment were analysed by using the Image program. The myocardial apoptosis was measured by terminal deoxynucleotidyl transferase dUTP nick end labeling (TUNEL) according to the manufacturer’s instructions (Roche Applied Science, South San Francisco, California, USA). Five micrographs were randomly selected and the numbers of healthy or apoptotic cardiomyocytes were counted. The extent of cell apoptosis was expressed as the ratio of TUNEL-positive nuclei over DAPI-stained nuclei.

### Animal model

Five weeks old female A/J mice were used. The mice were subcutaneously injected with 100 ul emulsion of 150 ug mouse RFP (Creative Biomart, USA) or supplemented Complete Freund’s Adjuvant (CFA) at the first and 7th day of the study. 3-weeks after the first injection of RFP, the mice were subjected to the LAD coronary ligation or sham operation as described previously [30]. Briefly, the mice were sedated with 2% isoflurane inhalation. Then the mouse precordial chest was incised between the 3rd and 4th rib to expose the heart after intubation and ventilation. 10/0 proline suture was passed under the LAD and ligated doubly. Finally, the chest wall was closed. Sham operation was done follow the same procedure without LAD ligation. All the animal work was approved by the Animal Care and Use Committee of the University of Heidelberg. The investigation conforms with the Guide for the Care and Use of Laboratory Animals published by the National Institutes of Health and was approved by the Government Presidium Karlsruhe (project No. 35–9185.81/G-168/12).

### Echocardiography and hemodynamic measurements

Echocardiography and hemodynamic measurements were performed under an anesthesia with 2% isoflurane. Cardiac function and morphology were determined in mice by echocardiography using a Visualsonics Vevo 770 system and a 30-MHz probe as described previously [31]. A Millar catheter-tip micromanometer catheter (SPR-671; Millar Instruments) connected to a Power Laboratory system (AD Instruments, Castle Hill, Australia) were used for the hemodynamic analysis as described previously [27, 29, 32].

### Measurement of autoantibody titers

Mouse plasma were collected and then were euthanized with carbon dioxide inhalation. Flat-bottomed 96-well microplates were coated with 100 pl /well of RFP at 5 pg/ml in bicarbonate buffer. The plates were incubated at 4 overnight. 300 ul of PBS containing 1% bovine serum albumin (BSA) were added into each well then incubated at room temperature for 1 h. After drying the plates on a pack of tissue towels, the plates were filled with 100 ul of diluted plasma samples in each well. The plasma samples were diluted in blocking buffer at different dilutions (1:250, 1:1250, 1:6250, 1:31250, 1:156250, 1:781250, 1:3906250). Then the plates were incubated with sealer for 3 hours at room temperature. After the removal of remaining liquid, the plates were filled with 10ul per well of peroxidase-conjugated goat anti-mouse secondary antibodies (Santa Cruz Biotechnology, USA), incubated for 2 hours at room temperature. Finally, the plates were filled with 100 ul per well of TMB peroxidase substrate and incubated about 15–20 min at room temperature. The colour reaction was stopped by adding 100 ul/well of 0.3 M sulfuric acid. The optical density values were determined by ELISA reader at 450–550 nm wavelength.

### Pathological test

Seven days after the LAD ligation, mice were euthanized and hearts was excised. After dissecting LV, part of myocardial samples was snap-frozen with liquid nitrogen for protein as well as mRNA analysis and the other part was fixed for 24 hours in 4% formalin dissolved in 0.1 M PBS (pH 7.4), subsequently embedded in paraffin, and transversely cut into 5 μm sections onto slides for further histological analysis. Sections were stained with hematoxylin and eosin (HE) to determine the myocyte cross-sectional area as described previously [31]. Myocardial infarction size was determined by morphometric analysis as described previously [30]. The myocardial apoptosis in the infarct border zone was measured by TUNEL [30].

### Western blotting

Total ventricular extracts were prepared from the LV myocardium as described previously [31]. Protein concentrations were measured by BCA (Sigma-Aldrich, USA) assay. Equal amount of protein extracts was separated with SDS-page and transferred to a nitrocellulose membrane (Millipore, USA). The following primary antibodies were used in the present study: anti-GAPDH (USBiological, USA), anti-FZD1 (R&D systems, USA), anti-active-β-catenin (Millipore, USA), anti-β-catenin (Santa Cruz Biotechnology, USA), anti-p-GSK-3β (Cell-Signaling Technology, USA), anti-GSK-3β (BD Biosciences, Germany). HRP-conjugated anti-rabbit IgG, anti-mouse IgG, and anti-goat IgG antibodies (Santa Cruz Biotechnology, USA) were used as secondary antibodies. The blots were analyzed and quantified by densitometry using Image J program.

### Quantitative real-time PCR

Total RNA was extracted from the left ventricles of the animals using Trizol (Invitrogen, USA), and first-strand cDNA was synthesized with the Revert Aid first strand cDNA synthesis Kit (Fermentas, USA). The cDNA was subjected to quantitative real-time PCR by Taqman probe analysis (Roth, Switzerland) performed using a LightCycler^®^ (Roth, Switzerland). All real-time PCR reactions were performed in triplicate and normalized to hypoxanthine phosphoribosyltransferase (HPRT) expression. The following primers were used in the present study: mouse HPRT forward 5′-GTCAACGGGGGACATAAAAG-3′, reverse 5′-TGCATTGTTTTACCAGTGTCAA-3′; mouse FZD1 forward 5′- CAGCAGTACAACGGCGAAC-3′, reverse 5′- GTCCTCCTGATTCGTGTGGC -3; mouse β- myosin heavy chain (β- MHC) forward 5′-CAAGGTCAATACTCTGACCAAGG-3′, reverse 5′-CCATGCGCACTTTCTTCTC-3′.

### Statistical analysis

Data are shown as mean ± SEM. Statistical analysis was performed with the Graph-Pad Prism Software Package Version 5.0 (GraphPad, Inc.). The one-way analysis of variance (ANOVA) was used to compare multiple groups, and the least square difference method (LSD) was applied to compare two groups. *P* < 0.05 was considered statistically significant.

## SUPPLEMENTARY MATERIALS FIGURES



## Abbreviations

BSA: bovine serum albumin
BW: body weight
CSA: cardiomyocyte surface area
DAPI: 4’,6-diamidino-2-phenylindole dihydrochloride
DMEM: dulbecco’s modified eagle medium
EF: ejection fraction
FCS: fetal calf serum
FZD1: frizzled1
GSK-3β: glycogen synthase kinase-3β
HR: heart rate
HW: heart weight
LAD: left anterior descending
LV: left ventricle
LVW: left ventricular weight
LW: lung weight
LVAWD: left ventricular anterior wall end-diastolic thickness
LVEDP: LV end-diastolic pressure
LVESP: LV end-systolic pressure
LVIDd: left ventricular internal end-diastolic dimensions
MI: myocardial infarction
NRCM: neonatal rat cardiomyocytes
RFP: recombinant FZD1 protein
TL: tibia length
α-MHC: α-myosin heavy chain
β-MHC: β-myosin heavy chain
