# Supplementary material for: Recombinant frizzled1 protein attenuated cardiac hypertrophy after myocardial infarction via the canonical Wnt signaling pathway
Source: Oncotarget. 2017 Dec 12;9(3):3069–80. doi: 10.18632/oncotarget.23149 (PMC5790446; doi:10.18632/oncotarget.23149)
Supplement: Supplementary file 1 [file oncotarget-09-3069-s001.pdf]

Recombinant frizzled1 protein attenuated cardiac hypertrophy after myocardial infarction via the canonical Wnt signaling pathway

SUPPLEMENTARY MATERIALS

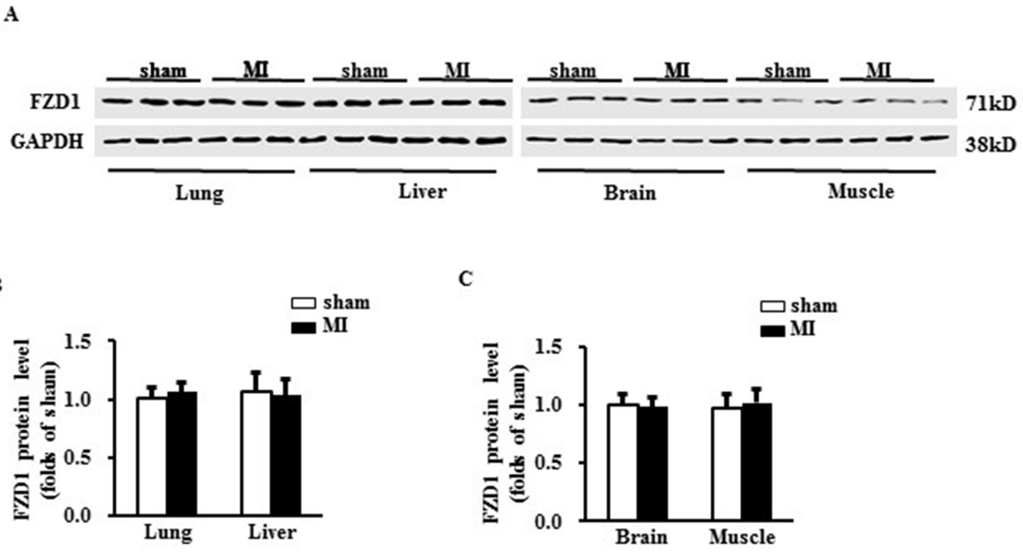

Supplementary Figure 1: (**A**) Representative western blots and (**B**, **C**) the quantitative result of the expression of FZD1 in the mouse left ventricle before and one week after LAD ligation. *N* = 5 independent experiments in all groups. All data are expressed as mean ± S.E.M.

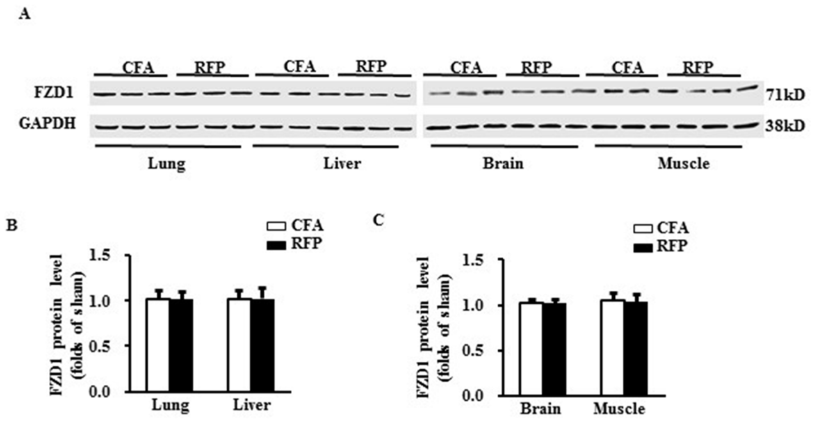

Supplementary Figure 2: (**A**) Representative western blots and (**B**, **C**) the quantitative result of the expression of FZD1 in theleft ventricles of the MI mice before and after the treatment of RFP. *N* = 5 independent experiments in all groups. All data are expressed as mean ± S.E.M.
